# Clinical and demographic profile of patients with keratoconus in the Eastern Cape, South Africa

**DOI:** 10.4102/jcmsa.v4i1.364

**Published:** 2026-08-19

**Authors:** Śruti M. Dave, Daniel S. Louw

**Affiliations:** 1 Department of Ophthalmology, Faculty of Medicine and Health Sciences, Walter Sisulu University, Gqeberha, South Africa

**Keywords:** keratoconus, demographics, prevalence, Africa, Eastern Cape

## Abstract

**Background:**

Keratoconus (KC) is a progressive corneal ectasia and a leading indication for corneal transplantation in South Africa. While prevalence data exist for other provinces, there is a paucity of literature describing the disorder profile in the Eastern Cape, a region characterised by unique socio-economic challenges and limited access to specialists.

**Methods:**

A retrospective chart review was conducted on 131 patients (262 eyes) diagnosed with KC at a specialist corneal practice in Gqeberha between September 2019 and September 2020. Demographics, visual acuity, KC severity and management markers were assessed using Galilei G6 tomography and clinical records.

**Results:**

The median age at assessment was 29 years (interquartile range: 21.5–40.0), with 62.6% of patients aged under 35 years. The cohort was racially diverse, comprising 41.22% black people, 38.17% white people, 14.50% people of mixed race and 6.11% Indian people. Mild and moderately severe disease was most common in both eyes, representing 64.12% of left eyes and 58.78% of right eyes. Severity was greater in the left eye and significantly associated with eye rubbing (*p* = 0.042). In all, 53.44% of patients were female. Corneal collagen cross-linking was recommended in 31.30% of left eyes, and 25.95% of patients qualified for keratoplasty.

**Conclusion:**

Patients in the Eastern Cape present with advanced KC at a young age. The high burden of severe pathology necessitates aggressive early detection and treatment strategies.

**Contribution:**

These findings support the need for screening and improved access to progression-halting and optical rehabilitation services to prevent reversible blindness in this economically active population.

## Introduction

Keratoconus (KC) is a non-inflammatory, bilateral corneal ectasia characterised by progressive thinning, irregular astigmatism and reduced visual function.^1^ It is the most common corneal ectasia^2^ and is well recognised as a multifactorial condition, with biomechanical, biochemical, environmental and genetic components contributing to the resultant corneal collagen changes. The disease typically manifests in the second decade of life, a critical period for education and economic productivity.^3^ It is well documented that younger patients, especially those diagnosed with KC before adulthood, are at an increasingly higher risk of progression and at an increased likelihood of requiring corneal transplantation later in life.^4,5^

In the South African public healthcare context, delayed diagnosis as a result of limited primary eye care infrastructure often results in preventable visual impairment.^6^ The economic implications are profound; young adults blinded by untreated ectasia lose their economic potential, placing a strain on social welfare systems. The prognosis of corneal transplantation in children is poorer than in adults.^7^ Therefore, a treatment to halt progression, corneal collagen cross-linking (CXL), before the condition progresses to the point of a corneal graft, is beneficial. Corneal collagen cross-linking has consistently been shown to decrease the progression of KC in multiple studies globally. Young patients, by inference, are the greatest beneficiaries of early interventions aimed at halting disease progression.

Global literature indicates significant variability in KC prevalence and severity across geographies and ethnicities.^8^ Sriranganathan et al. indicate a global KC prevalence of 0.24%.^8^ The highest rates are observed in Africa and South Asia,^8,9^ with prevalence increasing over time, particularly among younger age groups, particularly those aged 18–39 years, with peak incidence in the 20–29-year age group.^8,9,10,11^ The second-highest group was found to be among individuals aged 40–64 years.^10^ Men are more frequently affected in most global cohorts, and black women in the United States demonstrated a higher prevalence.^8,9,10,11^ In India, young men are disproportionately affected, with a substantial proportion of cases observed among students.^9^

In South Africa, studies from Gauteng and KwaZulu-Natal have documented distinct regional profiles, suggesting a younger age of onset and more aggressive disease in black African populations.^12,13^ In addition, black African women had a higher frequency of KC compared to men and other ethnic groups.^12,13^ Most studies highlight sparse population-based data and wide heterogeneity, with pooled estimates likely inflated by clinic-based samples.

The Eastern Cape, a province with a population of 7.2 million (2022 Census),^14^ has a vast rural catchment and historically under-resourced health infrastructure and remains a ‘data desert’ regarding corneal ectasia. No published data currently describe the demographic or clinical characteristics of KC patients in this region. This study aimed to bridge that knowledge gap. By establishing a baseline profile, this research allows for comparison with national data. It determines whether the Eastern Cape population exhibits unique phenotypic traits or severity patterns that warrant region-specific clinical protocols.

### Aim and objectives

This study aimed to describe the demographic and clinical profile of patients diagnosed with KC in the Eastern Cape. The specific objectives were to describe the distribution of KC severity and assess the association between the demographic profile and severity.

## Research methods and design

### Study design

A retrospective, descriptive chart review was conducted. This design was selected to facilitate the efficient analysis of existing clinical data and establish a ‘real-world’ baseline of the disease burden.

### Setting

The study was conducted at a specialist corneal practice in Gqeberha (formerly Port Elizabeth), Eastern Cape. This facility serves as a primary referral centre for corneal pathology for the western region of the Eastern Cape and the Garden Route district. The practice manages a diverse patient demographic, receiving referrals from both private optometrists and state facilities, thereby reflecting the broader burden of disease in the province.

### Study population and sampling strategy

The study population consisted of all patients diagnosed with KC who presented to the study site over a 13-month period, from 01 September 2019 to 30 September 2020. The selected period ensured the inclusion of all relevant cases and minimised seasonal and operational variations in case presentations. A census sampling strategy was employed, whereby all patients meeting the inclusion criteria during the study period were included. Inclusion criteria encompassed all patients with a confirmed diagnosis of KC, based on Scheimpflug tomography (Galilei G6, Ziemer Ophthalmic Systems, Switzerland) and clinical slit-lamp examination. Patients with other corneal ectasias, such as pellucid marginal degeneration or post-laser-assisted in situ keratomileusis (LASIK) ectasia, and those with incomplete clinical records were excluded from the study.

### Data collection

Data were extracted from electronic medical records (EMR) using a standardised data extraction sheet. Demographic variables captured included age at presentation, gender and observer-assigned ethnicity (classified as black, white, mixed-race or Indian). The medical history was reviewed for documentation of atopy (asthma, eczema), eye rubbing, a family history of KC, previous CXL, current contact lens usage and previous keratoplasty. Clinical metrics included uncorrected visual acuity (UCVA) and best corrected visual acuity (BCVA) measured with a phoropter, recorded in Snellen notation, and converted to decimal values for statistical analysis.

Tomographic indices from the Galilei G6, specifically keratometry (K) readings, maximum keratometry (Kmax) and thinnest corneal thickness (TCT), were used to confirm the diagnosis of KC. For statistical analysis, each eye was graded for severity into four severity groups according to the steepest K value in dioptres (D): mild (<45D), moderate (46D–52D), advanced (53D–59D) and severe (>59D).^15^ For statistical purposes, two patients diagnosed with forme fruste KC were included in the ‘mild’ group. Finally, the primary management modality recommended or performed was categorised into observation, contact lenses, CXL or keratoplasty.

### Data analysis

Data were collected in Microsoft Excel and analysed using Power BI and R (Version 4.4.2). Continuous variables, such as age, were tested for normality using the Shapiro-Wilk test. Normally distributed variables are presented as means with standard deviations (s.d.), while non-normally distributed variables are summarised as medians with interquartile ranges (IQR). Categorical variables are reported as frequencies and percentages.

For comparisons between two groups, the *t*-test was used for normally distributed variables, and the Mann-Whitney U test was applied for non-normally distributed variables. For comparisons involving more than two groups, analysis of variance (ANOVA) was used for normally distributed variables, while the Kruskal-Wallis test was used for non-normally distributed variables. The association between two categorical variables was assessed using Pearson’s chi-squared test when the expected frequency counts were five or more. When frequency counts were small (*n* < 5), Fisher’s exact test was used. A *p*-value less than 0.05 was considered statistically significant, while *p*-values between 0.05 and 0.10 were interpreted as indicating borderline trends.

### Ethical considerations

Ethical approval was obtained from the Walter Sisulu University Faculty of Health Sciences Human Research Ethics and Biosafety Committee (Ref: WSU HREC 427/2025). The study adhered to the principles outlined in the Declaration of Helsinki (2022). A waiver of informed consent was granted because of the retrospective nature of the research and the anonymisation of data prior to analysis. Strict confidentiality was maintained by assigning unique study identifiers to all records.

## Results

### Demographics

The study cohort comprised 131 patients. The demographic characteristics are summarised in Table 1. The median age at presentation was 29 years (IQR: 21.5–40.0). The age distribution was positively skewed towards younger adults, with 32.06% of the cohort in the 20–29 age group. The cohort was mostly female (*n* = 70; 53.44%) and black (*n* = 54; 41.22%).

**TABLE 1:** Demographic profile of the study cohort (*N* = 131).

Variable	Cohort	%
**Age (years)**		
< 20	26	19.85
20–29	42	32.06
30–39	27	20.61
≥ 40	36	27.48
**Gender**		
Female	70	53.44
Male	61	46.56
**Ethnicity**		
Black people	54	41.22
White people	50	38.17
Mixed-race people	19	14.50
Indian people	8	6.11

### Clinical presentation, management and risk profile

The clinical characteristics, management interventions and risk factor profile for the study cohort are summarised in Table 2. The visual acuity results demonstrate significant impairment; the median UCVA was 0.2 (decimal) in the right eye and 0.25 in the left eye. Best corrected visual acuity improved to a median of 0.8 bilaterally.

**TABLE 2:** Clinical profile of patients.

Variable	Right eye (*n* = 131)				Left eye (*n* = 131)				Combined or patient (*N* = 131)	
	Median	IQR	*n*	%	Median	IQR	*n*	%	*n*	%
**Visual acuity**										
Uncorrected (UCVA)	0.2	0.063–0.4	-	-	0.25	0.063–0.5	-	-	-	-
Best Corrected (BCVA)	0.8	0.32–1	-	-	0.8	0.32–1	-	-	-	-
**Medical history and risk factors**										
Previous CXL	-	-	2	1.5	-	-	3	2.3	11	8.4
Eye rubbing	-	-	-	-	-	-	-	-	71	54.2
Allergies	-	-	-	-	-	-	-	-	69	52.7
Asthma	-	-	-	-	-	-	-	-	15	11.5
Family history	-	-	-	-	-	-	-	-	11	8.4
Eczema	-	-	-	-	-	-	-	-	10	7.6
**Contact lens usage**										
RGP	-	-	-	-	-	-	-	-	26	19.9
Scleral	-	-	-	-	-	-	-	-	10	7.6
Hybrid	-	-	-	-	-	-	-	-	7	5.3
Hard	-	-	-	-	-	-	-	-	3	2.3
Soft	-	-	-	-	-	-	-	-	1	0.8
**Planned intervention**										
CXL recommended	-	-	36	27.5	-	-	41	31.3	-	-
PK or DALK planned	-	-	10	7.6	-	-	22	16.8	2	1.5

IQR, interquartile range; UCVA, uncorrected visual acuity; BVCA, best corrected visual acuity; CXL, corneal collagen cross-linking; RGP, rigid gas permeable; PK, penetrating keratoplasty; DALK, deep anterior lamellar keratoplasty.

Analysis of risk factors (Table 2) revealed a high prevalence of atopy and mechanical trauma. Eye rubbing was reported in 54.20% (*n* = 71) of patients and allergies in 52.6% (*n* = 69). A family history of KC was noted in 8.40% of the cohort. Corneal collagen cross-linking was recommended for 27.48% of right eyes and 31.30% of left eyes. Contact lens usage was common, with 19.85% of patients utilising rigid gas permeable (RGP) lenses.

### Factors associated with severity

**FIGURE 1:**
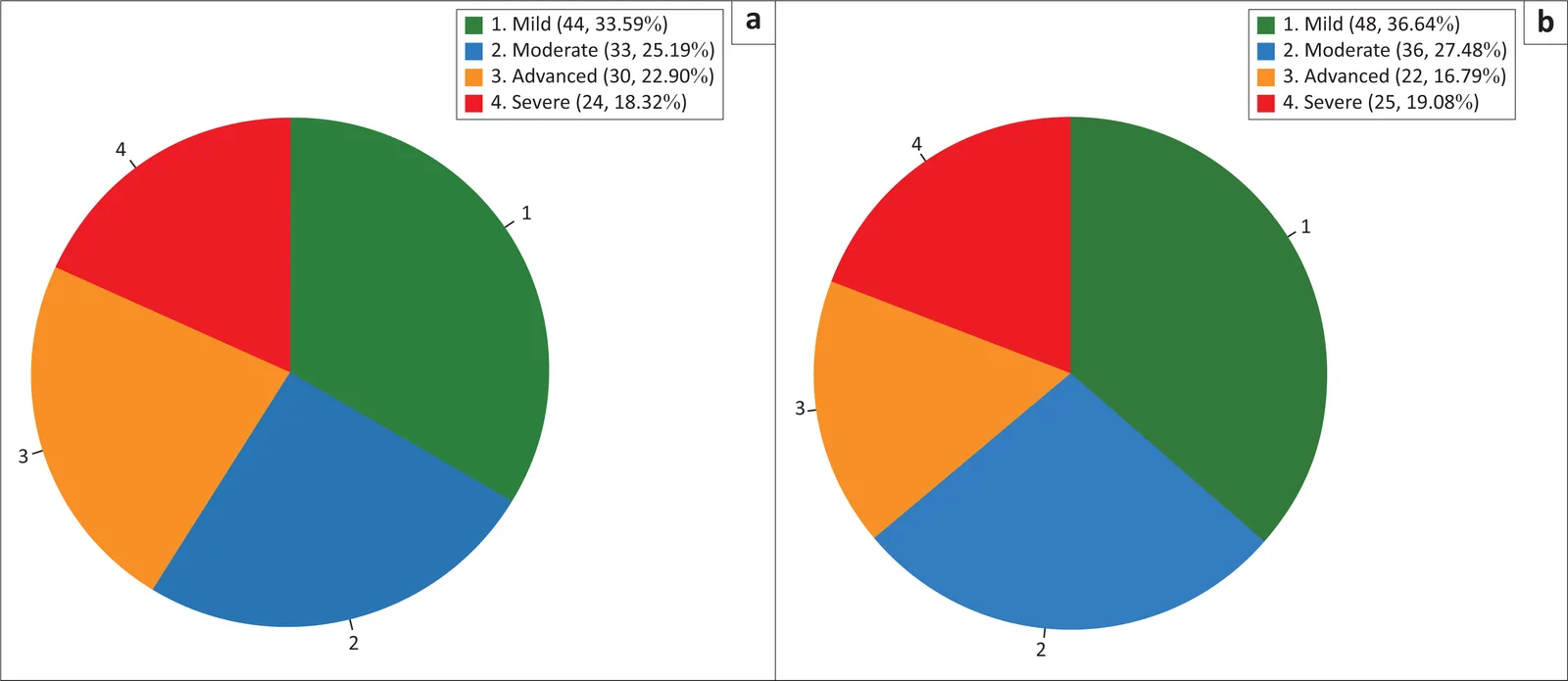
Distribution of severity for (a) right eye; and (b) left eye.

Disease severity was stratified into four categories: mild, moderate, advanced and severe (Figure 1). When adding up their representative percentages, mild and moderate disease combined was most common in both eyes, constituting 64.12% of left eyes and 58.78% of right eyes.

The associations between demographic, clinical and management variables with disease severity for both eyes are detailed in Table 3 and Table 4. In the right eye, age showed a borderline significant association with severity (*p* = 0.063), with older patients presenting with more severe disease. Gender also showed a borderline association (*p* = 0.058), with men comprising 70.8% of the ‘Severe’ group in the right eyes. In the left eyes, eye rubbing showed a statistically significant association with severity categories (*p* = 0.042). For both eyes, the use of contact lenses and the planning of penetrating keratoplasty or deep anterior lamellar keratoplasty (PK or DALK) were strongly confined to patients presenting with advanced disease severity (*p* < 0.001).

**TABLE 3:** Associations between demographics and keratoconus severity for left eyes.

Variable	Mild (*n* = 48)				Moderate (*n* = 36)				Advanced (*n* = 22)				Severe (*n* = 25)				*p*-value
	*n*	%	Median	IQR	*n*	%	Median	IQR	*n*	%	Median	IQR	*n*	%	Median	IQR	
**Age (years)**	-	-	26	19–50.2	-	-	29	22–35	-	-	30	25–37	-	-	37	26–46	0.238
Gender	-	-	-	-	-	-	-	-	-	-	-	-	-	-	-	-	0.876
Female	25	52.1	-	-	21	58.3	-	-	12	54.5	-	-	12	48.0	-	-	-
Male	23	47.9	-	-	15	41.7	-	-	10	45.5	-	-	13	52.0	-	-	-
**Race**	-	-	-	-	-	-	-	-	-	-	-	-	-	-	-	-	0.208
Black people	19	39.6	-	-	13	36.1	-	-	10	45.5	-	-	12	48.0	-	-	-
White people	25	52.1	-	-	12	33.3	-	-	5	22.7	-	-	8	32.0	-	-	-
Mixed-race people	3	6.2	-	-	7	19.4	-	-	5	22.7	-	-	4	16.0	-	-	-
Indian people	1	2.1	-	-	4	11.1	-	-	2	9.1	-	-	1	4.0	-	-	-
**Clinical history**																	
Eye rubbing	30	62.5	-	-	23	63.9	-	-	7	31.8	-	-	11	44.0	-	-	0.042*
Allergies	28	58.3	-	-	20	55.6	-	-	10	45.5	-	-	11	44.0	-	-	0.582
Asthma	8	16.7	-	-	1	2.8	-	-	3	13.6	-	-	3	12.0	-	-	0.254
Eczema	3	6.2	-	-	3	8.3	-	-	3	13.6	-	-	1	4.0	-	-	0.626
Family history	6	12.5	-	-	2	5.6	-	-	1	4.5	-	-	2	8.0	-	-	0.602
Previous CXL	2	4.2	-	-	4	11.1	-	-	5	22.7	-	-	5	20.0	-	-	0.085
Contact lenses use	5	10.4	-	-	12	33.3	-	-	13	59.1	-	-	17	68.0	-	-	< 0.001*
CXL recommended	15	34.1	-	-	17	51.5	-	-	6	20.0	-	-	3	12.5	-	-	0.007*
PK or DALK planned	1	2.1	-	-	2	5.6	-	-	15	68.2	-	-	16	64.0	-	-	< 0.001*

IQR, interquartile range; CXL, corneal collagen cross-linking; PK, penetrating keratoplasty; DALK, deep anterior lamellar keratoplasty.

* , significant *p*-values (*p* < 0.05).

**TABLE 4:** Associations between demographics and keratoconus severity for right eyes.

Variable	Mild (*n* = 44)				Moderate (*n* = 33)				Advanced (*n* = 30)				Severe (*n* = 24)				*p*-value
	*n*	%	Median	IQR	*n*	%	Median	IQR	*n*	%	Median	IQR	*n*	%	Median	IQR	
**Age**	-	-	25.5	17–42.2	-	-	27	22–35	-	-	32	23–37	-	-	41	25.8–53	0.063
**Gender**	-	-	-	-	-	-	-	-	-	-	-	-	-	-	-	-	0.058
Female	27	61.4	-	-	20	60.6	-	-	16	53.3	-	-	7	29.2	-	-	-
Male	17	38.6	-	-	13	39.4	-	-	14	46.7	-	-	17	70.8	-	-	-
**Race**	-	-	-	-	-	-	-	-	-	-	-	-	-	-	-	-	0.756
Black people	19	43.2	-	-	13	39.4	-	-	12	40.0	-	-	10	41.7	-	-	-
White people	20	45.5	-	-	10	30.3	-	-	12	40.0	-	-	8	33.3	-	-	-
Mixed-race people	3	6.8	-	-	8	24.2	-	-	4	13.3	-	-	4	16.7	-	-	-
Indian people	2	4.5	-	-	2	6.1	-	-	2	6.7	-	-	2	8.3	-	-	-
**Clinical history**																	
Eye rubbing	29	65.9	-	-	18	54.5	-	-	15	50.0	-	-	9	37.5	-	-	0.148
Allergies	27	61.4	-	-	16	48.5	-	-	18	60.0	-	-	8	33.3	-	-	0.121
Asthma	6	13.6	-	-	3	9.1	-	-	3	10.0	-	-	3	12.5	-	-	0.923
Eczema	4	9.1	-	-	1	3.3	-	-	2	6.7	-	-	3	12.5	-	-	0.757
Family history	6	13.6	-	-	3	9.1	-	-	1	3.3	-	-	1	4.2	-	-	0.369
Previous CXL	3	6.8	-	-	6	18.2	-	-	3	10.0	-	-	4	16.7	-	-	0.411
Contact lenses use	4	9.1	-	-	12	36.4	-	-	16	53.3	-	-	15	62.5	-	-	< 0.001*
CXL recommended	8	18.2	-	-	17	51.5	-	-	11	36.7	-	-	0	0.0	-	-	-
PK or DALK planned	2	4.5	-	-	5	15.2	-	-	16	53.3	-	-	11	45.8	-	-	< 0.001*

IQR, interquartile range; CXL, corneal collagen cross-linking; PK, penetrating keratoplasty; DALK, deep anterior lamellar keratoplasty.

* , significant *p*-values (*p* < 0.05).

## Discussion

### Key findings

This study establishes that KC in the Eastern Cape affects a diverse, young population (median 29 years). A large proportion of patients are diagnosed at advanced stages, with over 40% of right eyes classified as Advanced or Severe at presentation. While not statistically significant (*p* > 0.05), strong trends suggest that male gender and advancing age are associated with greater disease severity. High rates of atopy and eye rubbing were observed, consistent with known environmental risk factors and with prior meta-analyses.^16^ Visual function tracks the severity of disease strongly: worst eye BCVA falls markedly from mild and moderate (median 0.80) to advanced (0.25) and severe (0.12). Contact lens use at presentation is associated with greater disease severity, consistent with referral pathways and prior optical rehabilitation attempts.

### Discussion of key findings

The median age of 29 years in this Eastern Cape cohort aligns with findings from KwaZulu-Natal (median 24 years)^13^ but is notably older than in Middle Eastern cohorts, where presentation often occurs in the late teens.^17^ The combination of an ‘older’ young-adult demographic and severe pathology suggests a pattern of delayed diagnosis rather than late onset. In a well-resourced setting, these patients would likely have been identified in their late teens during routine optometric screening. In the Eastern Cape, structural barriers to eye care likely result in patients seeking help only when their visual function is significantly compromised.

The trend towards greater severity in men (*p* = 0.058) mirrors global literature suggesting that male patients often present with more advanced ectasia, potentially because of higher rates of vigorous eye rubbing or occupational exposure.^16,18^ The prevalence of eye rubbing (54.20%) and allergies (52.67%) (Table 2) in this cohort is substantial, reinforcing the mechanical pathway in KC pathogenesis and highlighting a modifiable risk factor for patient education.

In the left eyes, eye rubbing showed a statistically significant association with severity categories (*p* = 0.042). Jaśkiewicz found a relation between left eye severity and preference for left eye rubbing and left-handedness.^18^

Handedness was not documented by the treating clinician. As a result, no inference could be made regarding the association between handedness and the sidedness of KC severity in this study.

The association between older age and severity likely reflects the natural history of the untreated disease. In the absence of early intervention with CXL, ‘mild’ KC in a 20-year-old often progresses to ‘severe’ KC by age 40 years. This ‘progression by neglect’ is a hallmark of health systems in which preventive interventions are not universally accessible.

Contrary to historical perceptions of KC as a disease of specific ethnic groups, our data show a balanced distribution across black and white populations, with no significant difference in severity by race (*p* = 0.756). This supports the growing consensus that KC is a pan-ethnic condition in South Africa, necessitating the development of inclusive screening strategies.^12,13^

### Strengths and limitations

The main strengths of this study lie in the real-world evaluation of KC severity and its management. The use of high-resolution Scheimpflug tomography enhances diagnostic accuracy and facilitates effective treatment planning in this setting. It provides the first reliable dataset for the Eastern Cape region. However, limitations are acknowledged. The study was based in a private specialist practice, and data were collected during the recent coronavirus disease 2019 (COVID-19) pandemic. While the practice receives state referrals, indigent patients in rural areas who cannot afford transport to Gqeberha are likely under-represented. Consequently, the true burden of neglected, end-stage disease is likely higher than reported here.

### Implications or recommendations

The findings have two critical implications for public health policy in the Eastern Cape. Firstly, the reliance on patients to self-report vision loss is insufficient. Screening protocols utilising portable keratometry should be integrated into school health programmes to detect the disease in the ‘mild’ phase (teens and early 20s) before anatomical changes, including scarring, occur.

The unprecedented accumulation of deferred medical consultations resulting from the COVID-19 pandemic fuelled the development of artificial intelligence (AI)-powered telemedicine tools and platforms in healthcare, including ophthalmology. The deployment of AI and telemedicine enabled clinicians to manage a greater number of patients remotely, as part of a broader containment and mitigation strategy to reduce the need for face-to-face consultation. For KC, AI has been shown to predict both local and overall (global) patterns of progression based on Scheimpflug parameters, thereby enabling earlier treatment of patients at higher risk of keratoconic disease progression.^19^

Secondly, with a significant proportion of eyes requiring CXL (approx. 30%), this procedure cannot remain the purview of tertiary or private centres alone. Expanding access to CXL at regional levels is a cost-effective strategy to reduce the long-term state burden of corneal transplantation and disability grants.

## Conclusion

Keratoconus in the Eastern Cape is a condition primarily affecting young, economically active adults, characterised by late presentation and high rates of severe disease. The findings highlight a ‘silent epidemic’ of preventable blindness driven by delayed diagnosis and limited access to care. Over 23.7 million individuals globally are affected by KC.^10^ The increasing global burden of KC emphasises the need for further research into regional patterns to inform public health strategies and optimise patient care. Robust provincial epidemiology, long-term local outcomes and cost-effectiveness analyses are essential to guide policy and National Health Insurance (NHI) planning. Urgent investment in early detection via tomography-enabled hubs, sustained CXL services, funded public contact lens pathways and strengthened eye banking are required to alter the trajectory of the condition in this province and mitigate preventable visual impairment.
